# Characterization of the pathogenic α-Synuclein Variant V15A in Parkinson´s disease

**DOI:** 10.1038/s41531-023-00584-z

**Published:** 2023-10-30

**Authors:** Sokhna Haissatou Diaw, Max Borsche, Linn Streubel-Gallasch, Marija Dulovic-Mahlow, Julia Hermes, Insa Lenz, Philip Seibler, Christine Klein, Norbert Brüggemann, Melissa Vos, Katja Lohmann

**Affiliations:** 1 Institute of Neurogenetics, University of Lübeck, 23562 Lübeck, Germany; 2 Department of Neurology, University Hospital Schleswig Holstein, Lübeck, Germany

**Keywords:** Parkinson's disease, Mutation

## Abstract

Despite being a major component of Lewy bodies and Lewy neurites, pathogenic variants in the gene encoding alpha-Synuclein (α-Syn) are rare. To date, only four missense variants in the *SNCA* gene, encoding α-Syn have unequivocally been shown to be disease-causing. We here describe a Parkinson´s disease patient with early cognitive decline carrying an as yet not fully characterized variant in *SNCA* (NM_001146055: c.44T > C, p.V15A). We used different cellular models, including stably transfected neuroblastoma (SH-SY5Y) cell cultures, induced pluripotent stem cell (iPSC)-derived neuronal cultures, and generated a *Drosophila* model to elucidate the impact of the p.V15A variant on α-Syn function and aggregation properties compared to other known pathogenic variants. We demonstrate that p.V15A increased the aggregation potential of α-Syn and the levels of apoptotic markers, and impaired the mitochondrial network. Moreover, p.V15A affects the flying ability and survival of mutant flies. Thus, we provide supporting evidence for the pathogenicity of the p.V15A variant, suggesting its inclusion in genetic testing approaches.

## Introduction

Parkinson’s disease (PD) is characterized by dopaminergic neuronal loss in the pars compacta of the substantia nigra^1^. The primary neuropathological characteristic in most cases of PD is the abnormal aggregation of alpha-Synuclein (α-Syn) in the form of Lewy bodies and neurites^2^.

Variants in the *SNCA* gene, encoding α-Syn, were the first to be causally linked to rare autosomal dominantly inherited forms of PD^3^. Among all *SNCA* mutation carriers, the median age at onset of PD is 46 years, with 33% having an onset before 40 years and cognitive decline being a frequently observed feature^4^. The mutational spectrum includes whole gene triplications^5^, duplications^6,7^, and several missense variants, of which p.A53T^3^ and p.A30P^8^ are the best-characterized ones (www.mdsgene.org). It has been shown that most patients with *SNCA* triplications have more severe clinical symptoms, including earlier onset age and higher frequencies of psychosis and depression, and a faster disease progression compared to duplication and some carriers of missense variants^4^.

**Fig. 1:**
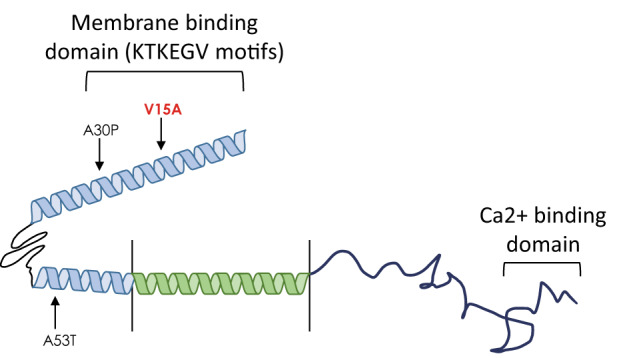
SNCA variant p.V15A and well-known pathogenic variants p.A30P and p.A53T on the SNCA protein. Schematic representation of alpha-Synuclein monomeric structure showing the positions of the newly identified missense variant p.V15A (NM_000345: c.44T>C) (in red), and the established variants p.A30P (NM_000345: c.88G>C) and p.A53T (NM_000345: c.157G>A). In light blue is the N-terminal region, in green is the NAC region, and in blue is the C-terminal region. The Figure is adapted from^67^ and^68^.

*SNCA* encodes a 140-residue protein comprised of three domains: an N-terminal amphipathic region (residues 1–60) where membrane recognition takes place, a central aggregation-prone region (residues 61–95) known as the non-Aβ component (NAC), and a negatively charged C-terminal domain where calcium binds (residues 96–140)^9^ (Fig. 1). α-Syn aggregates exhibit various structures, ranging from nonfibrillar oligomers to spherical protofibrils (leading to annular oligomers upon prolonged incubation) to insoluble fibrils^10–12^. Aggregates formed by these fibrils were shown to be the main component of Lewy bodies, one of the hallmarks of PD^13^. Additionally, mutant α-Syn appears to accumulate faster than oligomeric wildtype α-Syn. α-Syn aggregation and Lewy body pathology occur in many cell types like cortical neurons, dopaminergic neurons, and the enteric nervous system^14–17^. In human neuroblastoma cell lines and rat neuronal cultures, SNCA expression was shown to be cytotoxic at both low micromolar and overexpressed levels^18,19^. Furthermore, there is a threshold concentration above which wildtype α-Syn detrimental consequences emerge, and its overexpression of mutant and wildtype α-Syn seems to induce oxidative stress in cells^18^.

The N-terminal domain of α-Syn includes highly conserved KTKEGV hexameric motifs, which are important for α-Syn-lipid interactions and give α-Syn the ability to disrupt lipid bilayers. It is known that α-Syn interacts with lipids in its monomeric conformation, a form in which it regulates synaptic vesicle trafficking^20^. In addition, various forms of α-Syn can penetrate cells and create high molecular weight species, and α-Syn plasma membrane binding properties are crucial prior to its cell internalization^21^. Moreover, α-Syn localization and binding to mitochondria were reported in several studies^22–25^. However, the potential function of α-Syn in mitochondria is unclear since both loss and overexpression of α-Syn are proposed triggers of mitochondrial dysfunction^26–29^.

In *Drosophila melanogaster*, there is no *SNCA* orthologue. However, α-Syn models have been created by ectopically expressing wildtype or mutant forms of *SNCA*. The resulting α-Syn expression in such flies led to parkinsonian-like phenotypes, including loss of dopaminergic neurons and locomotion deficits^30,31^, indicating that flies can be used to model α-Syn-dependent PD.

In this study, we identified, at the time of detection, a previously unreported, potentially pathogenic missense *SNCA* variant (NM_001146055: c.44 T > C, p.V15A) in a 52-year-old patient from Germany of Turkish origin. Subsequently, this variant was reported in four siblings from an Italian family^32^ and in three affected individuals from two unrelated Japanese families^33^. Our functional analyses in different cell models and *Drosophila* indicate that p.V15A triggers α-Syn accumulation comparable to known pathogenic *SNCA* variants, as well as impairing the mitochondrial machinery. It further affects flying ability and survival in mutant flies. These findings strongly point to the fact that p.V15A is a PD-causing variant.

## Results

### Clinical presentation

We report a 52-year-old male index patient from a non-consanguineous family of Turkish origin. Before the diagnosis of PD, he was suffering from myocardial infarction at the age of 40 years. After a therapeutic intervention, he developed delirium with disorientation, hallucinations, and aggressive behavior. In the following, he continued experiencing anxiety and panic attacks; thus, a psychiatric disorder was suspected. At age 41 years, the patient developed a resting tremor of the left upper limb. Three years later, rigidity, predominantly on the left side, appeared, together with bradykinesia and gait problems. Upon neurological examination, the patient was diagnosed with PD and received dopaminergic treatment resulting initially in a significant improvement of motor symptoms. The patient reported having never been on antipsychotic medication before being diagnosed with PD. However, from the time point of diagnosis, he was on quetiapine because of psychotic syndromes related to dopaminergic treatment. Additionally, severe cognitive impairment and motor fluctuations that occurred early during the disease course prevented him from continuing his work.

At the age of 47 years, the patient was under treatment with levodopa/carbidopa/entacapone 150/37.5/200 mg five times a day, safinamide 50 mg daily, and clozapine 12.5 mg per day. In the OFF state, he exhibited slight dysarthria, mild hypomimia, and rigidity of the left upper extremity. Finger tapping and alternating pronation-supination movements were severely slowed and irregular in both hands, again pronounced on the left side. The gait was slow and impaired, with reduced stride length and an increased number of turning steps, but he could still walk unaided. Freezing was present at gait initiation but not during continuous walking. He presented impaired postural stability, global slowness, poverty of spontaneous movements, and resting tremor of all limbs but no head tremor. He scored 54/138 on the MDS-UPDRS III; the Hoehn and Yahr stage was 3. He suffered from OFF periods as well as from dyskinesia, both with a relevant functional impact. He could not perform various activities of daily living most of the time and was severely limited from participating in social interactions.

Handwriting and orthostatic function were normal, but he had anosmia (BSIT: 0/12), while hyposmia was reported to have begun in childhood and worsened over time. The patient additionally reported constipation, apathy, and fatigue (nM-EDL score of 42/48 points). Sleep was severely disturbed, objectified by a PDSS-2 score of 55/60 points, and daily sleepiness was strikingly increased (ESS: 17/24 points). However, the patient’s daily and family life was most severely affected by psychiatric symptoms like anxiety and depression, notified by scoring high on both scales of the HADS questionnaire (Anxiety score: 19/21 points; Depression score: 16/21 points). Intermittently, he experienced severe delusions and paranoia. Moreover, severe cognitive impairment was detected by 17/30 points in the MoCA score. At the age of 52 years, he was a wheelchair user, severely demented and illegible due to hypophonia and dysarthria.

His mother was diagnosed with PD at the age of 50 years, exhibiting asymmetric resting tremor, dysarthria, dysphagia, and gait disturbance. Later during the disease course, she developed dementia. She died at the age of 57 years from pneumonia. His father, at the time of examination 75 years old, was described as having a mild tremor upon agitation. Among his three older sisters, two are reportedly healthy, while the third sister was diagnosed with panic attacks.

### Genetic Analysis

GenePanel analysis covering 29 PD and dystonia genes revealed, at that time, a novel missense variant in *SNCA* (NM_000345.3:c.44T > C, p.V15A, Supplementary Fig. 1, ClinVar ID: 871101). Family members were not available to test for segregation. While the variant was not listed in >140,000 individuals in gnomAD v2 (https://gnomad.broadinstitute.org/gene/ENSG00000145335?dataset=gnomad_r2_1), it has been found in a single individual of European (non-Finnish) decent in gnomAD v3.1 (https://gnomad.broadinstitute.org/variant/4-89835624-A-G?dataset=gnomad_r3). The variant has a CADD score of 28.2 (V1.6; https://cadd.gs.washington.edu/). The amino acid substitution p.V15A is located within the N-terminal amphipathic domain (Fig. 1). This region is not only responsible for membrane binding, but it is also where other pathogenic variants linked to familial PD like p.A53T and p.A30P are located^9^. Therefore, the variant was scored initially as a variant of uncertain significance (VUS) thus prompting functional characterization^34^.

### α-Syn p.V15A, p.A30P, p.A53T expression in stably transfected neuroblastoma cell lines

**Fig. 2:**
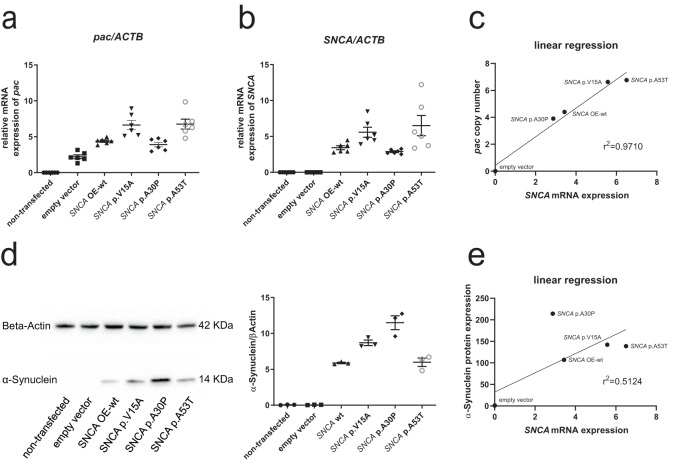
α-Syn p.V15A properties in transfected neuroblastoma cell lines. The quantification of (**a**) *pac* and (**b**) *SNCA* mRNA levels in neuroblastoma (SH-SY5Y) cell lines from a non-transfected line, a line with an empty vector, and lines stably transfected with wildtype (wt) *SNCA*, p.A30P, p.V15A, and p.A53T. The results are based on the mean ratios of the target genes’ expression compared to the reference gene *ACTB*. Differences were analysed using one-way analysis of variance (ANOVA) with a Tukey post-hoc test. All *p*-values were calculated using SNCA OE-wt as a control group: (**a**) p = 0.0107 for *SNCA* p.V15A; *p* = 0.9631 for *SNCA* p.A30P; *p* = 0.0060 for *SNCA* p.A53T; (**b**) *p* = 0.2205 for *SNCA* p.V15A; *p* = 0.9902 for *SNCA* p.A30P; *p* = 0.0265 for *SNCA* p.A53T. Means and standard error of the mean (SEM) (*n* ≥ 3 independent experiments) are indicated. **c** Correlation of the expression levels was tested using linear regression analysis, including *pac* copy numbers and *SNCA* gene expression as variables. The regression line and coefficient of determination (r^2^) are indicated. The axes are labeled with arbitrary values (only relative quantifications were performed). **d** Western blot analysis of total protein extract from SH-SY5Y cell lines from a non-transfected line, a line with an empty vector, and lines overexpressing wildtype (wt) SNCA, p.A30P, p.V15A, and p.A53T with antibodies against α-Syn and β‐actin (loading control) under basal condition. Differences were analysed using one-way analysis of variance (ANOVA) with a Tukey post-hoc test. All *p*-values were calculated using SNCA OE-wt as a control group (*p* = 0.0153 for *SNCA* p.V15A; *p* < 0.0001 for *SNCA* p.A30P; *p* > 0.9999 for *SNCA* p.A53T). Means and standard error of the mean (SEM) (*n* ≥ 3 independent experiments) are indicated. **e** Correlation of expression was tested using linear regression analysis, including SNCA protein expression and *SNCA* mRNA expression as variables. The regression line and coefficient of determination (r^2^) are indicated.

After detecting this VUS, we wanted to elucidate its potential pathogenic effect further, starting with a human neuroblastoma cell line (SH-SY5Y). Since it is known that the disease mechanism of pathogenic variants in *SNCA* is related to increased protein accumulation, we first tested the accumulation properties of the p.V15A variant compared to the well-established p.A30P and p.A53T pathogenic variants in neuroblastoma cell lines. For this, we used cells stably overexpressing different forms of α-Syn: wildtype (OE-wt SNCA), or one of the following mutant forms p.V15A (c.44T > C), p.A30P (c.88 G > C), and p.A53T (c.157 G > A). The presence of the variants was confirmed by sequencing. In the first step, we evaluated the transfection efficiency by quantitative PCR (qPCR) of the *Puromycin-resistance* (*pac*) gene located on the vector. We demonstrated an increased presence of the *pac* gene in transfected cell lines demonstrating successful transfection. Further, we found varying levels of the *pac* gene, indicating that a different number of vectors had been included in the cell lines (Fig. 2a). In the next step, we measured the *SNCA* mRNA expression in each cell line. We also observed differences in *SNCA* expression which correlated with the amount of vector, indicating that the *SNCA* variants did not impact the mRNA expression level of *SNCA*, but the number of gene copies did as expected (Fig. 2b, c).

Since it is known that the α-Syn protein is the pathogenic agent, we next measured protein levels of the three mutants and the wildtype form using western blot analysis. Notably, protein expression levels did not correlate with the mRNA expression level indicating accumulation of some of the mutant proteins. The cell line with the p.A30P variant had the lowest expression on mRNA level (least *SNCA* gene copies) but showed the highest expression on the protein level, suggesting increased protein stability/decreased degradation. In this test, the p.V15A variant behaved similarly to the p.A53T variant, both of which had protein levels between those of the wildtype and the p.A30P variant (Fig. 2d, e).

### Activation of the apoptotic marker CI. Caspase 3 upon overexpression of α-Syn p.V15A in neuroblastoma cells

**Fig. 3:**
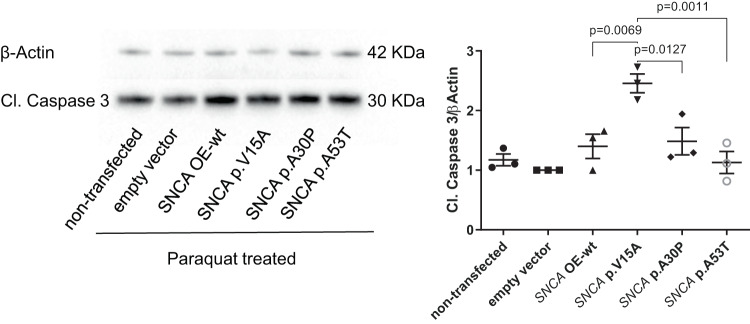
Activation of the apoptotic marker CI. Caspase 3 upon overexpression of α-Syn p.V15A in neuroblastoma cells. Western blot analysis of total protein extract from neuroblastoma (SH-SY5Y) cell lines from a non-transfected line, a line with an empty vector, and lines overexpressing wildtype (OE-wt) SNCA, p.A30P, p.V15A, and p.A53T with antibodies against Cl. Caspase 3 and β‐actin upon treatment with 2 mM of paraquat for 24 h. Data analysis was carried out for all western blots with an empty vector set as 1. Differences were analysed using one-way analysis of variance (ANOVA) with a Tukey post-hoc test. The mean, the standard error of the mean (SEM), and the *p*-values (*n* = 3 independent experiments) are indicated.

Several studies proved that in aggregates, wildtype α-Syn exerts a neural cell line-specific anti-apoptotic property, which seems to be lost by the p.A53T, but not by the p.A30P variant^35,36^. Given that Cleaved Caspase 3 (Cl. Caspase 3) is a frequently activated death protease^37–39^, we decided to monitor its protein expression in our overexpressing *SNCA* SH-SY5Y lines. Under basal conditions, we observed no significant difference in Cl. Caspase 3 protein levels between OE-wt and *SNCA* mutations (Supplementary Fig. 2). Paraquat (N,N-dimethyl-4-4′-bipiridinium) is a toxic chemical that decreases mitochondrial membrane potential, increases intracellular reactive oxygen species (ROS) levels, and induces apoptosis^40^. Therefore, we treated all cell lines with paraquat and observed increased levels of Cl. Caspase 3 protein, in p.V15A overexpressing cell lines compared to all other cell lines (Fig. 3), despite it having comparable α-Syn protein levels to OE-wt and p.A53T, and lower protein levels to p.A30P (Fig. 2d, e). Contrary to the literature, we observed upon paraquat treatment higher protein levels of Cl. Caspase 3 in p.A30P overexpressing cell lines compared to the p.A53T lines, suggesting a loss of α-Syn anti-apoptotic property in the p.A30P rather than the p.A53T variant.

### p.V15A has comparable aggregation properties to p.A53T in dopaminergic neurons

**Fig. 4:**
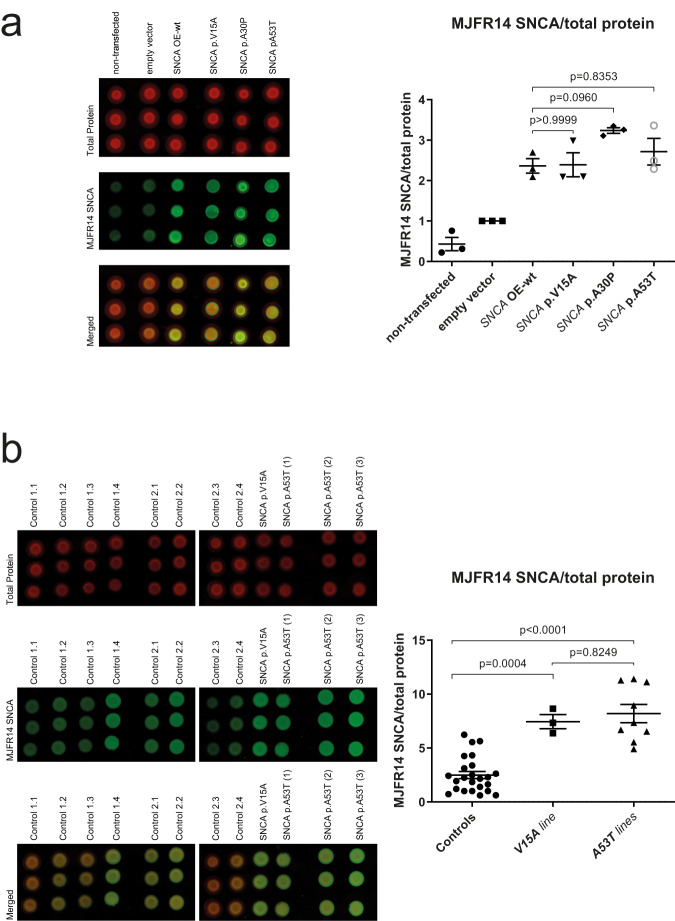
α-Syn p.V15A has comparable aggregation properties to p.A53T in neuroblastoma cell lines and in dopaminergic neurons. Native dot blot analysis of total protein extract from (**a**) neuroblastoma (SH-SY5Y) cell lines from a non-transfected line, a line with an empty vector, and lines overexpressing wildtype (OE-wt) *SNCA*, p.A30P, p.V15A, and p.A53T. Statistical significance in comparison to OE-wt. Data analysis was carried out for all dot blots with an empty vector set as 1. Each dot represents a biological replicate, and it is the mean value of a technical triplicate experiment. Differences were analysed using one-way analysis of variance (ANOVA) with a Tukey post-hoc test. The mean and the standard error of mean (SEM) are indicated. Native dot blot analysis of total protein extract from (**b**) iPSC-derived dopaminergic neuronal cell lines from four control lines 86-day-old (Control1.1-Control1.4), same four control lines 100-day-old (Control2.1-Control2.4) and 90-day-old lines expressing p.V15A and p.A53T. Statistical significance of multiple comparisons between the control group, V15A cell line, and A53T group. Data analysis was carried out for all dot blots with control 1.1 set as 1. Each dot represents a biological replicate, and it is the mean value of a technical triplicate experiment. Differences were analysed using one-way analysis of variance (ANOVA) with a Tukey post-hoc test. The mean, the standard error of mean (SEM), and the *p*-values (*n* ≥ 3 independent experiments) are indicated.

It has previously been shown that smaller protofibrils are a more toxic species of α-Syn causing neuronal cell death compared to larger amyloid fibrils^41–43^ and that the pathology may be associated with the number of oligomers as well as the structural and functional properties of the species formed^44^. To test for α-Syn aggregates in the neuroblastoma cell lines, we quantified α-Syn aggregates via native dot blot analysis using the recombinant Anti-Alpha-synuclein aggregate-specific antibody [MJFR-14-6-4-2], which binds selectivity to oligomers and aggregated α-Syn^45^. This demonstrated more signal/aggregates in the neuroblastoma lines overexpressing wildtype or mutant *SNCA* with a trend for the most pronounced aggregation in the line with the p.A30P variant (Fig. 4a) corresponding to the highest total α-Syn level (Fig. 2d).

Since transfection experiments, as done in the neuroblastoma cells, always result in overexpression at different levels for the different mutant forms of *SNCA* (Fig. 2b, d), we next wanted to study aggregation properties of mutant and wildtype α-Syn on the endogenous level. For this, we generated induced pluripotent stem cell (iPSC)-derived dopaminergic neurons from patients with the p.V15A (*n* = 1), and the p.A53T (*n* = 3) variant, respectively, and from four control lines with the wildtype form of *SNCA*. Native dot blot analysis revealed a significantly higher presence of aggregated α-Syn in all lines from carriers of *SNCA* variants compared to individuals with wildtype *SNCA* (Fig. 4b).

### Altered mitochondrial network in transfected neuroblastoma cell lines

**Fig. 5:**
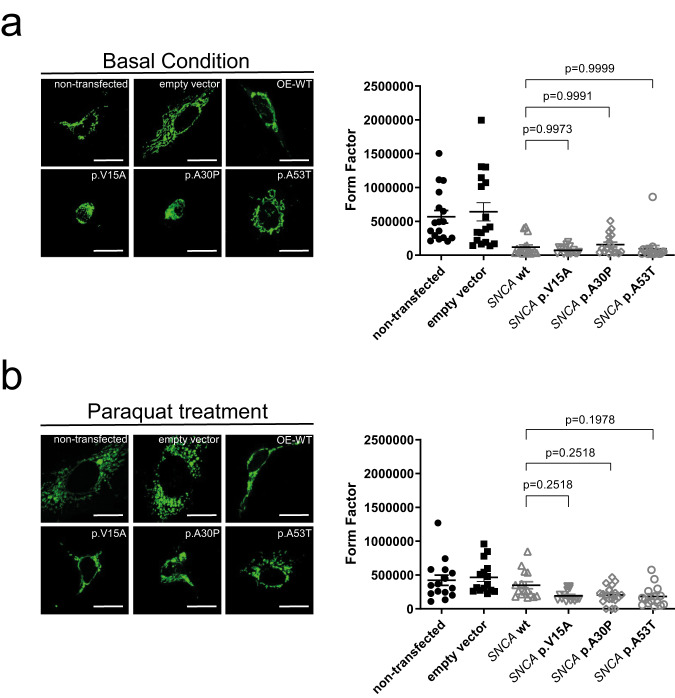
Altered mitochondrial network in transfected neuroblastoma cell lines. The mitochondrial network was visualized by confocal microscopy in fixed cells immuno-stained with anti-GRP75 (green) (**a**) under basal conditions and (**b**) upon treatment with 2 mM of paraquat for 24 h. A mean form factor (15–17 cells per cell line) was calculated as a measure of mitochondrial interconnectivity by using ImageJ (NIH software). Each dot represents the value of a single cell. The scale bar corresponds to 20 µm. Differences were analysed using one-way analysis of variance (ANOVA) with a Tukey post-hoc test. The means, standard error of the mean (SEM), and the *p*-values are indicated.

α-Syn is known to interact with synaptic vesicle membranes and native mitochondrial membranes, as shown in living cells^22,24,46,47^. The N-terminus of α-Syn, which contains the here-studied variants, is a crucial factor for mitochondrial membrane permeability regulation and is most likely a factor in neurodegeneration associated with PD^46^. Therefore, we also investigated the integrity of the mitochondrial network in wildtype and mutant transfected neuroblastoma cell lines. For this, we stained the mitochondrial network using a GRP75 antibody and calculated the form factor^48^. This showed an intact, interconnected, and branched tubular network in the non-transfected and empty vector-transfected lines as opposed to the small, separated tubules in the mutant SH-SY5Y lines indicating disturbed mitochondrial network integrity upon *SNCA* overexpression (comparison of the groups of non-*SNCA*-overexpressing lines vs. all *SNCA*-overexpressing lines with *p* < 0.0001 using an Unpaired t-test with Welch’s correction). Of note, the impact on the mitochondrial network seems to be related solely to the overexpression of *SNCA*, with no recognizable difference between wildtype and mutant forms (Fig. 5a).

Upon paraquat treatment, which triggers chronic, low-level oxidative stress in mitochondria, the transfected lines overexpressing *SNCA* exhibit signs of excessive accumulated damage with further isolated mitochondrial fragments. In addition, compared to the non-transfected and empty vector-transfected lines, they appear to have decreased mitochondrial branching and interconnectivity (Fig. 5b). Of note, the mutant lines seem to behave worse (lower form factor) when compared to wildtype overexpression (*p* = 0.0201 on the group level using an Unpaired *t*-test with Welch’s correction).

### Increased lethality rate and diminished locomotion in Drosophila melanogaster

**Fig. 6:**
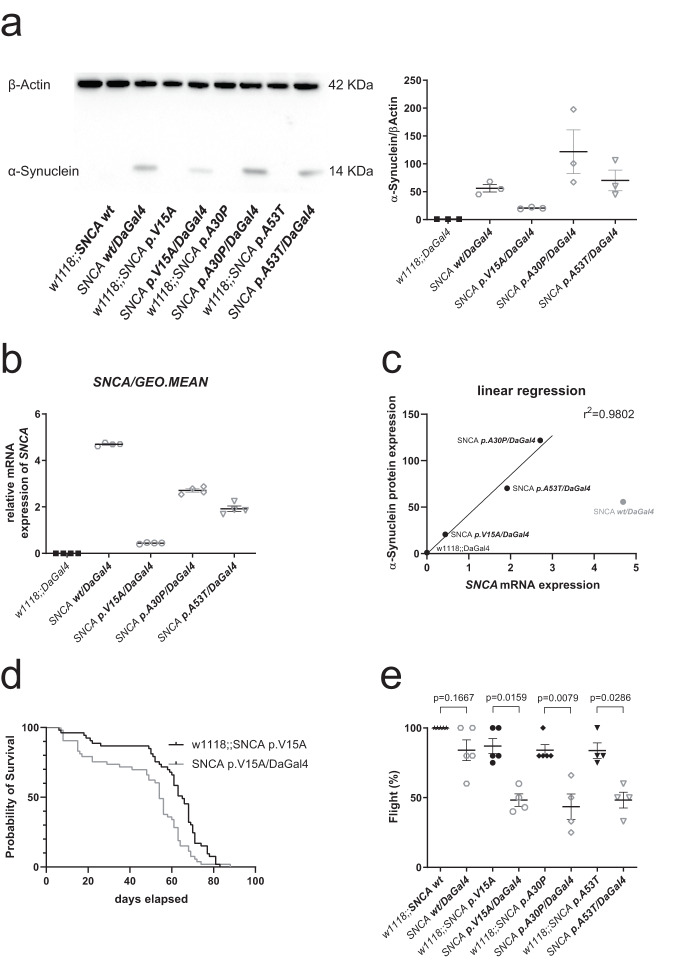
Parkinsonian-like phenotypes in flies overexpressing different human *SNCA* variants. Western blot analysis of total protein extract from 1-week-old *Drosophila* lines expressing human *SNCA* with antibodies against (**a**) α-Syn and β‐actin (loading control). **b** Quantification of SNCA mRNA levels in *Drosophila* lines expressing human *SNCA*. The results are based on the mean ratios of the target gene’s expression compared to the geometric mean of the reference genes *Act5c*, *EF1a2* and *RPL32*. Means and standard error of mean (SEM) (*n* ≥ 3 independent experiments) are indicated. Quantification per reference gene is illustrated in Supplementary Fig. 3. **c** Correlation of expression was tested using linear regression analysis, including SNCA protein expression and SNCA mRNA expression as variables in *Drosophila* lines expressing human *SNCA*. The outlier point SNCA wt/DaGal4 is represented in grey. The regression line and coefficient of determination (r2) are indicated (**d**) Survival rate is indicated for control flies (w1118;;SNCA p.V15A) and the hSNCA expressing flies with the driver (SNCA p.V15A/DaGal4). A minimum of 50 flies were tested (survival rates of other SNCA variants can be found in Supplementary Fig. 4). **e** Flying ability as a read-out for locomotion for control flies compared to α-Syn-expressing flies (with DaGal4) for the different α-Syn variants. Differences were analysed using Mann-Whitney test. The percentages, the standard error of the mean (SEM), and the *p*-values (*n* ≥ 3 independent experiments) are indicated.

To further evaluate the role of the p.V15A variant on the level of an organism, we created a *Drosophila* model expressing the human p.V15A α-Syn variant in the flies using the ubiquitous driver DaGal4. In addition, we included already available human α-Syn variants in our evaluation, namely, α-Syn wildtype, p.A30P, and p.A53T. The expression of the different α-Syn variants was confirmed via western blot and qPCR analyses following ubiquitous expression using the DaGal4 system compared to no expression when the driver was absent (Fig. 6a, b, Supplementary Fig. 3). Interestingly, the observed protein expression levels did only align with the corresponding mRNA expression levels for the mutant forms of SNCA mut/DaGal4 but not for SNCA wt/DaGal4 (Fig. 6c). Despite exhibiting the highest mRNA expression, this line displayed the lowest protein expression. This discrepancy suggests post-transcriptional regulatory mechanisms, such as protein degradation, translation efficiency, or protein stability, which may differ between the wildtype and mutant lines, further underlining the relevance of all three variants. Furthermore, we evaluated survival rates and observed that the expression of all α-Syn variants resulted in a decreased survival rate compared to their respective controls (Fig. 6d and Supplementary Fig. 4). Finally, we analyzed the flying ability of 3-week-old flies as a read-out for locomotion. All α-Syn expressing flies showed lower flying ability; however, not all to the same degree compared to their respective controls (Fig. 6e). Notably, for all three mutant α-Syn flies, the flying ability was significantly reduced also in comparison to the WT α-Syn expressing flies, while a heterozygous driver control did not induce any effects (data not shown). Thus, our data show that p.V15A expression in flies results in increased lethality and locomotion-defects.

## Discussion

While α-Syn makes up the majority of Lewy bodies, there are relatively few pathogenic variants identified in this gene. In this study, we present a patient with early-onset PD who experienced early cognitive and psychotic symptoms and rapid disease progression carrying a relatively novel, not fully characterized variant in the *SNCA* gene (p.V15A). However, based on the data provided within this manuscript and in the recent literature, the variant can now be considered as pathogenic and no longer as a VUS since the ACMG criteria PS3, PM1, and PP1-4 are fulfilled^32–34,49^. Notably, the previous family studies on patients with the p.V15A variant have reported clinical manifestations including motor dyskinesia, gait disturbance, dementia with Lewy bodies, and cognitive decline^32,33^ similar to other pathogenic variants in the *SNCA* gene^4^. Furthermore, individuals in both the Italian family and the Japanese study displayed positive responses to levodopa, resembling cases associated with sporadic late-onset Parkinson’s disease^32,33^.To evaluate the pathogenic role of the p.V15A in α-Syn, we established two cellular models and compared different read-outs: (1) SH-SY5Y cells overexpressing wildtype or mutant (p.V15A, p.A30P, p.A53T) *SNCA*, and used a non-transfected line and a line transfected with an empty vector as controls; (2) dopaminergic neurons derived from iPSCs from individuals with mutant (p.V15A, p.A53T) or wildtype α-Syn (controls). Finally, we generated a *Drosophila* model expressing human wildtype or different mutant *SNCA* including p.V15A (hSNCA-V15A).

In this study, we explored the integrity of several pathways that have been implicated in the disease mechanism of PD based on three genome-wide association studies (GWAS), including ER stress response, oxidative stress response, and immune response, with lipids or lipoproteins being critical to all processes^50^. It was found in a yeast study that α-Syn toxicity is not dependent on fibril formation and that toxicity is caused by interaction with cell membranes^51^. Furthermore, a more recent study in yeast demonstrated that α-Syn toxicity is correlated with the accumulation of lipid vesicles, which triggers ER to Golgi trafficking defects, suggesting that neurons’ dependence on lipid vesicle transport may be why they are so vulnerable to α-Syn toxicity^52^. Indeed, a recent study reports that compared to the wildtype, p.V15A triggers a helix-breaking effect and exhibits a lower affinity to the phospholipid membrane. Additionally, they observed more vigorous propagation activity as molecular effects of p.V15A α-Syn variant on PD’s pathogenesis compared to wildtype protein^33^.

In addition, these responses affect various cellular signaling processes, including apoptosis. Sequential activation of caspases plays a central role in the execution phase of cell apoptosis. In addition, some studies have revealed that Cl. Caspase 3 is essential for cell death and some of the characteristic changes in cell morphology and certain biochemical events associated with the execution and finalization of apoptosis^37,38,53^. Notably, we here showed in p.V15A overexpressing neuroblastoma cell line increased the level of Cl. Caspase 3 compared to all neuroblastoma lines. The results hint at a possible enhanced activation of Cl. Caspase 3’s apoptotic properties to recoup for α-Syn accumulation.

Overexpression of α-Syn in multiple cell types, including neurons, can lead to the fragmentation of mitochondria^54,55^, not by preventing fusion but by promoting mitochondrial fission^54^. In vitro, membranes containing the mitochondrial phospholipid cardiolipin can be fragmented by oligomerized α-Syn^54^. Interestingly, β- and γ-synuclein can also affect mitochondrial morphology, although to a lesser extent than α-Syn^54^, which suggests the involvement of the N-terminal membrane-binding domain, which is highly conserved between the three synuclein isoforms. When α-Syn binds to lipids, the N-terminus, which includes the here-studied variants, takes on an α-helical conformation, playing a critical role in its interaction with the membrane. A previous study suggested that the N terminus of α-Syn plays a significant role in regulating the permeability of mitochondrial membranes, and it is highly probable that it contributes to the neurodegeneration linked with PD^46^. Specifically, V15A and A30P are situated on the hydrophobic side of the first amphipathic helix, which is more important for phospholipid binding than the second amphipathic helix^56,57^. As aforementioned, lipids and lipoproteins functions are critical in the disease mechanism of PD. Based on our observations, we hypothesized that p.V15A (located in the N-terminal region) might trigger a dysfunctional mitochondrial network. In line with this, we exposed the neuroblastoma lines to paraquat, which triggers chronic, low-level oxidative stress in mitochondria. We observed with immunostaining analysis a non-significant reduced degree of mitochondrial branching in the p.V15A line compared to wildtype α-Syn after treatment with paraquat.

Abnormal α-Syn aggregation is a focal neuropathological characteristic of PD. We here explored the molecular effects of V15A mutation on α-Syn pathogenesis and, via native dot blot analysis, observed in dopaminergic neurons strong aggregation properties for V15A, comparable to the known variant A53T and stronger than the controls expressing wildtype *SNCA*.

The overexpression of human α-Syn p.V15A results in decreased survival rates and defects in locomotion, thereby recapitulating phenotypes that were previously described for the overexpression of other α-Syn variants and that we also confirmed here. Thus, we generated a α-Syn fly model that can be used to further investigate the underlying mechanisms. Interestingly, our data suggest that the p.V15A is less lethal than the other α-Syn variants, which can be explained by the relatively lower expression levels of p.V15A compared to the other α-Syn variants. Nonetheless, the locomotion defects, which is represented by the flying ability show similar severity, compared to the other mutant forms of α-Syn, whereas the overexpression of wildtype α-Syn appears less affected.

Altogether, our data and cumulative evidence from previous studies indicate that the p.V15A mutation has molecular and cellular effects comparable to those of the well-known variants p.A30P and p.A53T and is sufficient for α-Syn aggregation to occur. Our findings also underline the importance of additional pathogenic missense changes in this gene beyond the ones at the well-established positions p.A30 and p.A53. This needs to be accounted for in comprehensive genetic testing that should include gene sequencing – in addition to quantitative analyses - to uncover any hidden potentially pathogenic variant in PD patients. The identification of patients with pathogenic *SNCA* variants also has implications for personalized medicine since potential gene-specific therapies appear on the horizon^58,59^.

## Methods

### Generation of SH-SY5Y neuroblastoma cells and dopaminergic neurons

To assess the pathogenicity of p.V15A, we generated SH-SY5Y neuroblastoma cell models in which the variants p.V15A, p.A30P, p.A53T, and the wildtype were overexpressed. Lentiviral transduction was used to stably integrate the four SNCA constructs wildtype, p.V15A, p.A30P, p.A53T, and the pCSCW empty vector in SH-SY5Y. The integration of the SNCA constructs was confirmed by Sanger sequencing using BigDyeTM Terminator v3.1 (ThermoFisher) on 3500xL Genetic Analyzer. The overexpression of SNCA in the transduced SH-SY5Y cells was confirmed by quantitative PCR analysis with SYBR Green (Roche Diagnostics) on a LightCycler480 (Roche Diagnostics) using Maxima SYBR Green/Fluorescein qPCR Master Mix (2X) (Thermo Scientific). The housekeeping gene *ACTB* was used as a reference gene for normalizing the expression data. Additional confirmation was obtained by western blot analysis (anti-α-Syn antibody (1:1000 #2647; Cell Signaling) and anti-β-Actin (1:1000000 #A5316; Sigma)).

SH-SY5Y cells were maintained in Dulbecco’s modified Eagle’s medium (DMEM, Thermo Scientific, Waltham, MA, USA) supplemented with 10% fetal bovine serum (Life Technologies) and 1% penicillin/streptomycin (Life Technologies). Cells were cultivated at 37 °C and 5% CO2 in a humidified atmosphere.

To assay cells upon apoptosis induction, cells were treated with 2 mM of paraquat (Aldrich Chemistry) for 24 h.

Patient skin fibroblasts carrying the p.V15A variant were reprogrammed into iPSCs using Sendai virus to deliver the four reprogramming factors OCT4, SOX2, KLF4, and cMYC (CytoTune-iPS 2.0 Sendai Reprogramming Kit, Thermo Fisher Scientific). One iPSC clone was established according to the manufacturer’s protocol and cultured on Matrigel-coated dishes (BD Biosciences) in mTeSR1 medium (STEMCELL Technologies). The line was characterized for the expression of pluripotency markers and spontaneous differentiation potential to form embryoid bodies as described previously^60^ (Supplementary Fig. 5). A normal karyotype as well as the absence of large chromosomal aberrations was confirmed by single polymorphic nucleotide (SNP) profiling. Genome integrity was assessed by Illumina GSA-24v3.0 beadchip array and analyzed with GenomeStudio software (Illumina).

iPSC lines from four control individuals SFC084-03 (https://hpscreg.eu/cell-line/STBCi033-B), SFC086-03 (https://hpscreg.eu/cell-line/STBCi052-C), SFC089-03 (https://hpscreg.eu/cell-line/STBCi053-A), SFC156-03 (https://hpscreg.eu/cell-line/STBCi101-A) and three patient lines carrying the p.A53T mutation (SFC828-03, SFC829-03, SFC830-03) were generated and characterized previously^61^. The direct differentiation of iPSCs into dopaminergic neurons was conducted as described before^62,63^.

Neurons from control lines were harvested at day 86 (*n* = 4) and day 100 (*n* = 4), respectively; patient-derived neurons were harvested at day 90 of differentiation (*n* = 4). The cell lines’ details are listed in Table 1.

**Table 1:** Overview on individuals from whom iPSC cell lines were used in this study.

	Sex	Origin	Diagnosis	*SNCA* Genotype	Age of Onset	Age of donor (at collection)
L-11198	Male	Asian	PD	V15A/wt	40	48
SFC828-03	Female	European	PD	A53T/wt	39	51
SFC829-03	Male	European	PD	A53T/wt	40	46
SFC830-03	Male	European	PD	A53T/wt	42	51
SFC084-03	Female	European	Healthy control	wt/wt	/	63
SFC086-03	Female	European	Healthy control	wt/wt	/	57
SFC089-03	Female	European	Healthy control	wt/wt	/	64
SFC156-03	Male	European	Healthy control	wt/wt	/	74

*PD* Parkinson’s Disease, *Wt* wildtype, ages are given in years.

### RNA extraction and quantitative PCR analysis

According to the manufacturers’ instructions, total RNA from cell pellets (≈8.0 × 10^6^ cells) and drosophila (20 flies) was isolated using the RNeasy Plus Mini Kit (Qiagen) and the Monarch Total RNA MiniPrep Kit (NEB), respectively. 500 ng of RNA was a template for reverse transcription (First Strand cDNA Synthesis Kit, Thermo Scientific). Quantitative real-time PCR (qRT-PCR) was performed on the Lightcycler 96 (Roche Diagnostics) using Maxima SYBR Green/Fluorescein qPCR Master Mix (2X) (Thermo Scientific). The housekeeping gene *ACTB* was used as the reference genes for normalizing the expression data for cells. In the case of drosophila, the reference genes employed for normalizing the expression data were *dAct5c*, *deEF1a2*, and *dRPL32*. The primers used for qRT-PCR analysis are listed in Table 2.

**Table 2:** Sequences of primers used for qRT-PCR.

Housekeeping gene (qRT-PCR)	FORWARD (5’-3’)	REVERSE (5’-3’)
ACTB	TGAAGTGTGACGTGGACATC	GGAGGAGCAATGATCTTGAT
dAct5c	TCCACGAGACCACCTACAAC	CACTTGCGGTGCACAATGGA
deEF1a2	GCGTGGGTTTGTGATCAGTT	GATCTTCTCCTTGCCCATCC
dRPL32	ATCGGTTACGGATCGAACAA	GACAATCTCCTTGCGCTTCT

Target gene (qRT-PCR)		
pac	GAGTACAAGCCCACGGTGC	TTCTTGCAGCTCGGTGACC
SNCA	GGAGTTGTGGCTGCTGCTG	CACCACTGCTCCTCCAACAT

### Western blot analysis

Total protein was extracted from cell pellets using RIPA Lysis buffer (25 mM Tris–HCl pH 7.6, 150 mM NaCl, 1% DOC, 1% NP-40, 0.1% SDS, and proteinase inhibitor cocktail), measured with the DC Protein Assay (Bio-Rad) and gels were blotted onto nitrocellulose membranes. Primary antibodies used overnight at 4 °C for immunoblotting were as follows: anti- α-Syn (1:1000 #2647 for cells; 1:600 #2642 for flies; Cell Signaling), anti-Cleaved Caspase-3 (1:1000 #9662; Cell Signaling), and anti-β-Actin (1:1000000 #A5316; Sigma). Secondary antibodies used for 1 h at room temperature for immunoblotting were as follows: anti-Rabbit IgG, HRP-linked antibody (1:2500; Cell Signaling) and anti-Mouse IgG, HRP-linked antibody (1:2000; Cell Signaling). All western blots presented in the same figure derived from the same experiment and were processed in parallel. In general, blots for replication experiments were processed under comparable conditions.

### Native dot blot analysis

Total protein was extracted from cell pellets using RIPA Lysis buffer (25 mM Tris–HCl pH 7.6, 150 mM NaCl, 1% DOC, 1% NP-40, 0.1% SDS, and proteinase inhibitor cocktail). Protein extracts were used for the DC Protein Assay (Bio-Rad), and 7.5 μg of total protein extracts per sample were subjected to native dot blot analyses as previously described^64^. Total protein staining Revert 700 Stain Solution (Li-COR) was used as a loading control. Antibody signal intensities were normalized to the total protein. Antibodies used for the blotting analysis were as follows: primary antibody MJFR-14-6-4-2 (1:2000 #ab209538; Abcam) overnight at 4 °C and secondary antibody IRDye 800CW Goat anti-Rabbit (1:20000; Li-COR) for 1 h at room temperature. Detection and digitalization were performed using Odyssey CLx Infrared Imaging System (Li-COR). All the native dot blots originated from the same experiment and were processed in parallel.

### Immunofluorescence staining and image analysis

Cells were fixed on coverslips with 4% formaldehyde for 15 min at room temperature. Upon permeabilization, the cells were blocked with 0.1% Triton X-100 in 4% normal goat serum in PBS for 1 h. Immunofluorescence staining was carried out with primary antibody against GRP75 (1:1000 # ab53098; Abcam) overnight at 4 °C. Secondary fluorescence antibody Goat anti-Rabbit IgG Alexa Fluor 488 (1:400; Invitrogen), was applied for 1 h at room temperature. The cells were mounted with DAPI-Fluoromount-G® (Southern Biotech), and the images were taken as Z-stacks using a confocal microscope. At least fifteen cells from two coverslips per individual were analysed using ImageJ (NIH software; Rasband, W.S., ImageJ, U. S. National Institute of Health, Bethesda, Maryland, USA, https://imagej.nih.gov/ij/, 1997–2018) as described previously^48^.

### Generation of mutant flies

SNCA wildtype (w;;P{w[+mc]=UAS-Hsap\SNCA.F}5B), SNCA p.A30P (w;;P{w[+mc]=UAS-Hsap\SNCA.A30P}40.1), SNCA p.A53T (w;;P{w[+mc]=UAS-Hsap\SNCA.A53T}CG7900[15.3]), w1118 and dautherlessGal4 (DaGal4) were purchased from Bloomington stock Center (Indiana, USA). UAS-SNCA^V15A^ (SNCA p.V15A) was generated by insertion of the cDNA into the pUAST-attB overexpression vector^65^ via the NEBuilder Hifi DNA assembly cloning kit following the manufacturer’s protocol. The cDNA was obtained from the patient and cloned into the pUAST-attB vector using forward (TTGGGAATTCGTTAACAGATCTCAAAATGGATGTATTCATGAAAGGAC) and reverse (CCCTCGAGCCGCGGCCGCAGATCTTAGGCTTCAGGTTCGTAGT) primers and BglII restriction site. The SNCA p.V15A-containing vector was microinjected in the embryos of fly line *y1w1118;PBac{y*+*-attP-3B}VK00033* (stock number 9750 from the Bloomington stock center) by BestGene (USA). The flies carrying the construct were confirmed via sequencing and this construct (and the other SNCA variants) was ubiquitously activated via the DaGal4 driver.

### Survival rate and flying ability

Flies were divided in groups of ten male flies and every 2–3 days survival was documented. The flies were flipped to fresh medium every week. A minimum of fifty flies were analyzed for each genotype.

For flying ability, male flies were selected per group of five flies and placed in a cylinder. A flying score of 1 was given to those flies that were able to fly following the tapping of the cylinder. Those that were not able to fly or fell down were scored 0^66^. Data were presented in percentages. A minimum of 4 independent experiments were performed.

### Statistical analysis

Differences were analysed using analysis of variance (ANOVA) with a Tukey post-hoc test, Mann–Whitney test, or linear regression analysis. The error bars indicate the standard error of mean (SEM) of *n* > 3 independent experiments. Statistical differences were determined by *p*-values.

### Reporting summary

Further information on research design is available in the Nature Research Reporting Summary linked to this article.

### Supplementary information

Supplementary material

Reporting summary

## Data Availability

The authors confirm that the data supporting the findings of this study are available within the article and its supplementary materials.
