# Supplementary material for: Characterization of the pathogenic α-Synuclein Variant V15A in Parkinson´s disease
Source: NPJ Parkinsons Dis. 2023 Oct 30;9:148. doi: 10.1038/s41531-023-00584-z (PMC10616187; doi:10.1038/s41531-023-00584-z)
Supplement: Supplementary file 1 — Supplementary material [file 41531_2023_584_MOESM1_ESM.pdf]

**Supplemental figure 1**

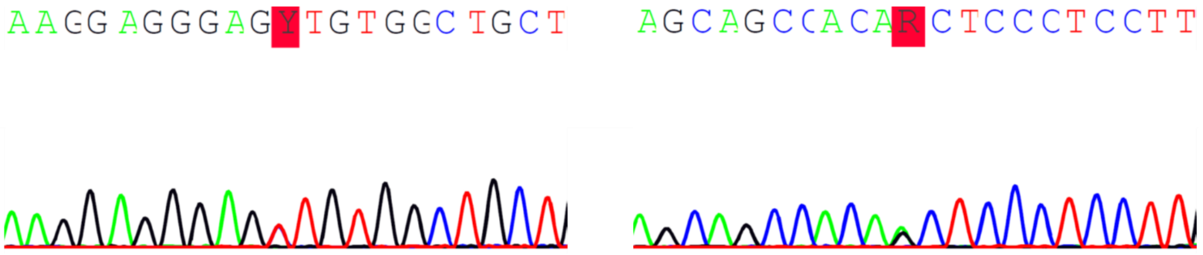


**Supplemental figure 1:** Electropherogram from Sanger sequencing of the c.44T>C variant in *SNCA*. The variant is shown in the forward (left panel) and reverse (right panel) direction and highlighted by the red label indicating two different alleles (wildtype and mutant) at the position c.44 in *SNCA* in the patient.

**Supplemental figure 2**

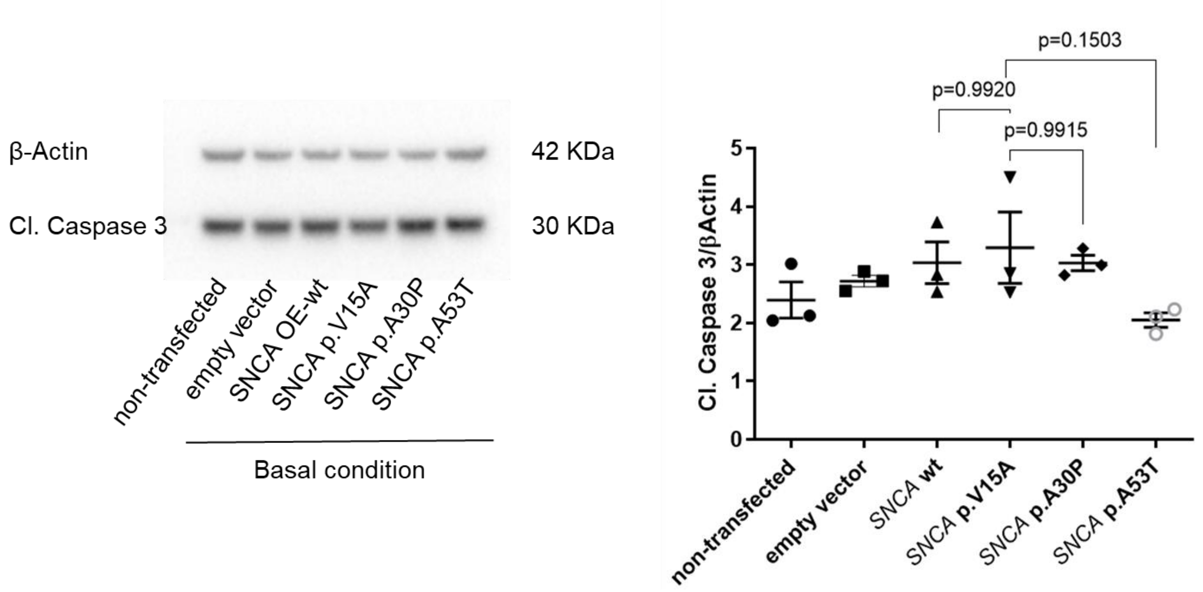


**Supplemental figure 2:** Western blot analysis of total protein extract from neuroblastoma (SH-SY5Y) cell lines from a non-transfected line, a line with an empty vector, and lines overexpressing wildtype SNCA, p.A30P, p.V15A, and p.A53T with antibodies against Cl. Caspase 3, Phospho-α-Synuclein and β-actin under basal conditions. Differences were analysed using one-way analysis of variance (ANOVA) with a Tukey' multiple comparisons post-hoc test. The mean, the standard error of mean (SEM), and the p-values (n=3 independent experiments) are indicated.

**Supplemental figure 3**

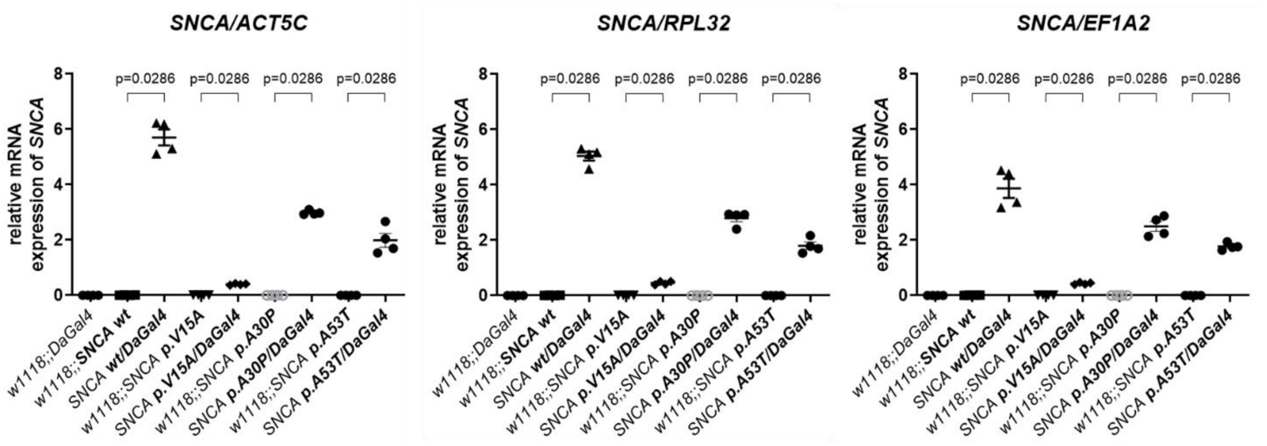


**Supplemental figure 3:** Quantification of *SNCA* mRNA levels in *Drosophila* lines expressing human *SNCA*. The results are based on the mean ratios of the target gene's expression normalized to the reference genes *Act5c*, *RPL32, and EF1a2*. Differences were analysed using Mann-Whitney test (all p-values<0.05). Means and standard error of mean (SEM) (n≥3 independent experiments) are indicated.

## Supplemental figure 4

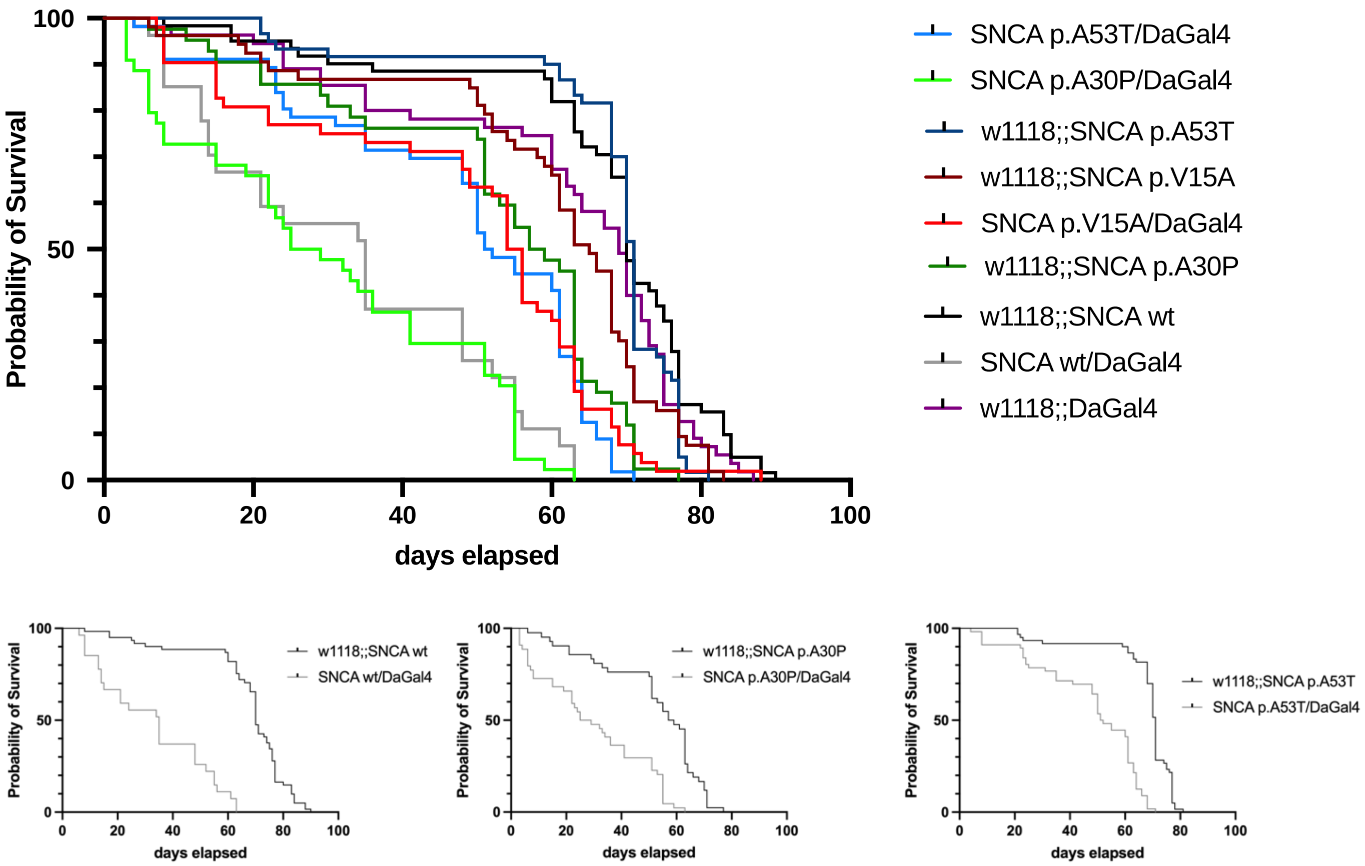


**Supplemental figure 4:** Survival rate is decreased upon expression of hSNCA. Survival probability is presented for all tested flies (upper panel) as well as for SNCA wildtype (wt), SNCA p.A30P and SNCA p.A53T (lower panel, left to right). The survival curves for V15A files are included in Figure 7d. A minimum of fifty flies were tested.

**Supplemental figure 5**

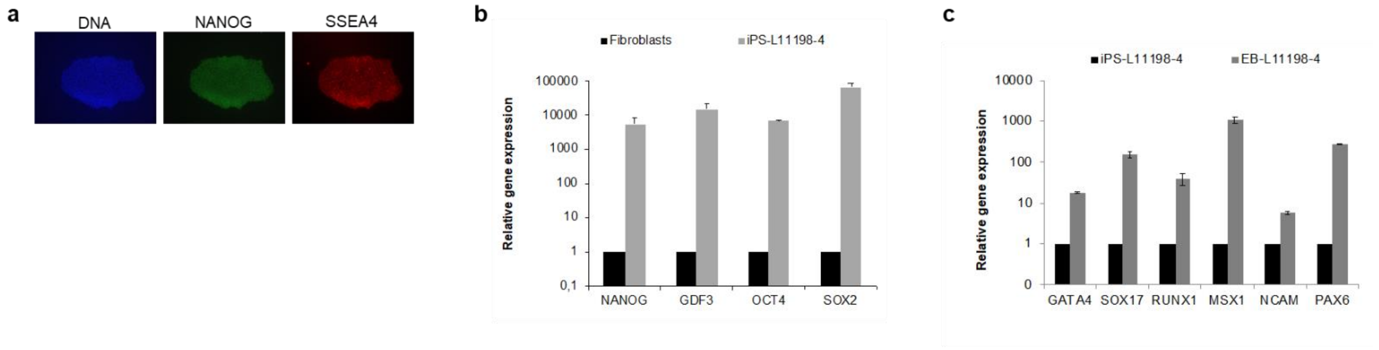


**Supplemental figure 5:** Characterization of line iPS-L11198-4. (a) Immunofluorescence analysis shows presence of pluripotency markers NANOG and SSEA4. (b) Expression levels of pluripotency markers NANOG, GDF3, OCT4, and SOX2 in fibroblasts and iPSCs relative to beta-actin (a loading control) as assessed by quantitative RT-PCR. The values from parental fibroblasts were set to 1. (c) Spontaneous differentiation potential via embryoid body (EB) formation. RT-PCR analyses of various differentiation markers for the three germ layers (endoderm: GATA4, SOX17; mesoderm: RUNX1, MSX1; ectoderm: NCAM, PAX6) in iPSCs that were undifferentiated (iPS-L11198-4) and after 14 days in culture (EB-L11198-4). The error bars indicate the standard deviations (SD).

**Supplemental table 1: Summary of all experiments performed in this study.**

| Models \ Experiments | | RT-qPCR | Western blot | Dot blot | Immuno-staining | Survival rate | Flying ability |
|---|---|---|---|---|---|---|---|
| Neuroblastoma cell lines | non-transfected | X | X | X | X | | |
| | empty vector | X | X | X | X | | |
| | SNCA wt | X | X | X | X | | |
| | SNCA p.V15A | X | X | X | X | | |
| | SNCA p.A30P | X | X | X | X | | |
| | SNCA p.A53T | X | X | X | X | | |
| iPSC-derived dopaminergic neuronal cell lines | SNCA wt (1.1) | | | X | | | |
| | SNCA wt (1.2) | | | X | | | |
| | SNCA wt (1.3) | | | X | | | |
| | SNCA wt (1.4) | | | X | | | |
| | SNCA wt (2.1) | | | X | | | |
| | SNCA wt (2.2) | | | X | | | |
| | SNCA wt (2.3) | | | X | | | |
| | SNCA wt (2.4) | | | X | | | |
| | SNCA p.V15A | | | X | | | |
| | SNCA p.A53T (1) | | | X | | | |
| | SNCA p.A53T (2) | | | X | | | |
| | SNCA p.A53T (3) | | | X | | | |
| Drosophila | W1118 | X | X | | | X | X |
| | wt | X | X | | | X | X |
| | wt+DaGal4 | X | X | | | X | X |
| | V15A | X | X | | | X | X |
| | V15A+DaGal4 | X | X | | | X | X |
| | A30P | X | X | | | X | X |
| | A30P+DaGal4 | X | X | | | X | X |
| | A53T | X | X | | | X | X |
| | A53T+DaGal4 | X | X | | | X | X |

## Uncropped western blots

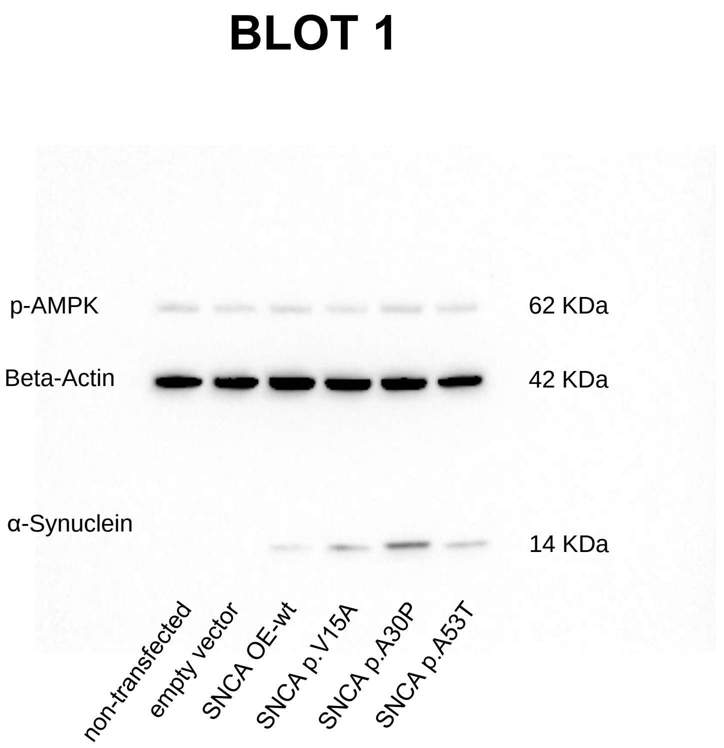


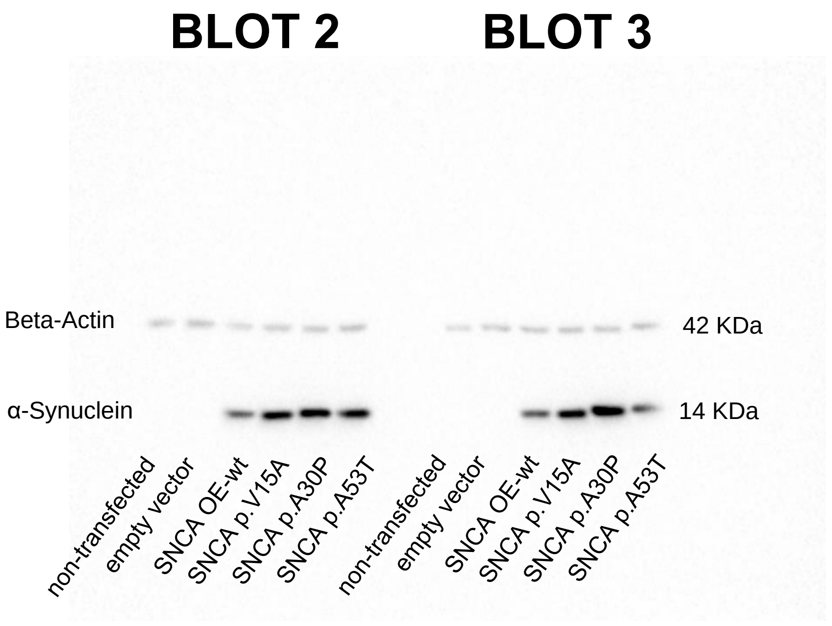


**Uncropped western blots corresponding to Figure 2: α-Syn p.V15A properties in transfected neuroblastoma cell lines.**

**BLOT 1**

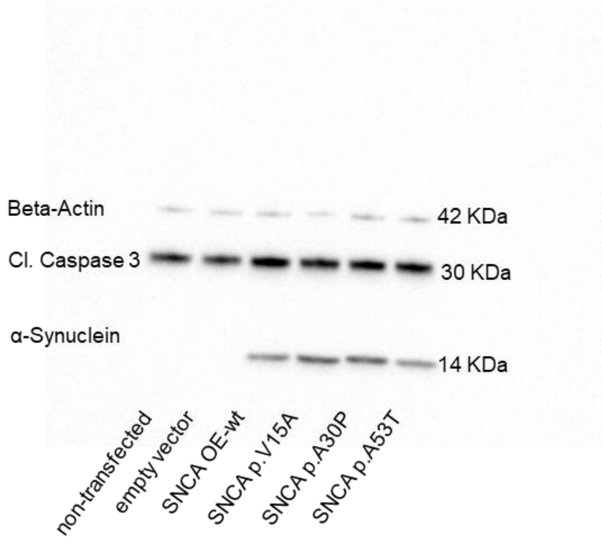


**BLOT 2**

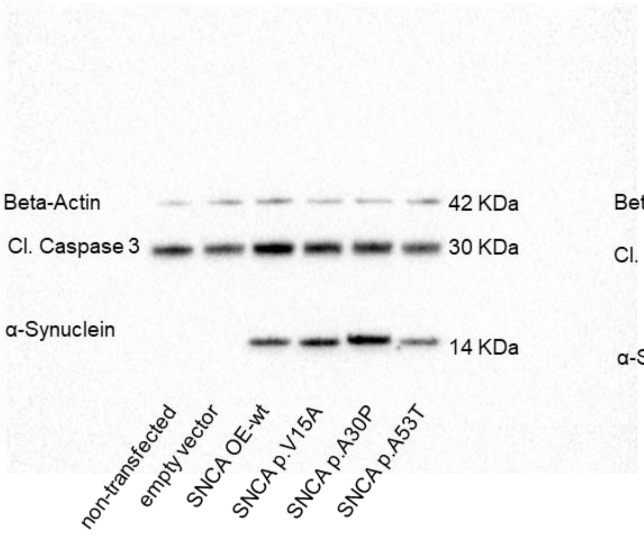


**BLOT 3**

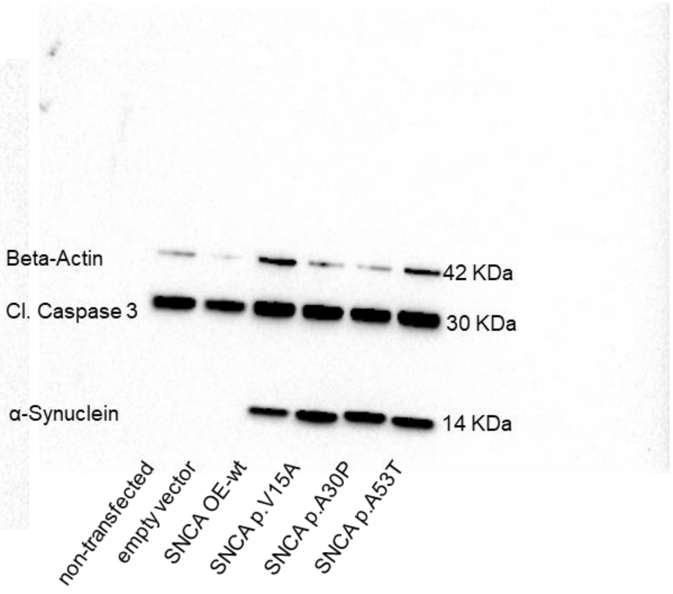


**Uncropped western blots corresponding to Figure 3: Activation of the apoptotic marker Cl. Caspase 3 upon overexpression of α-Syn p.V15A in neuroblastoma cells.**

**BLOT 1** **BLOT 2** **BLOT 3**

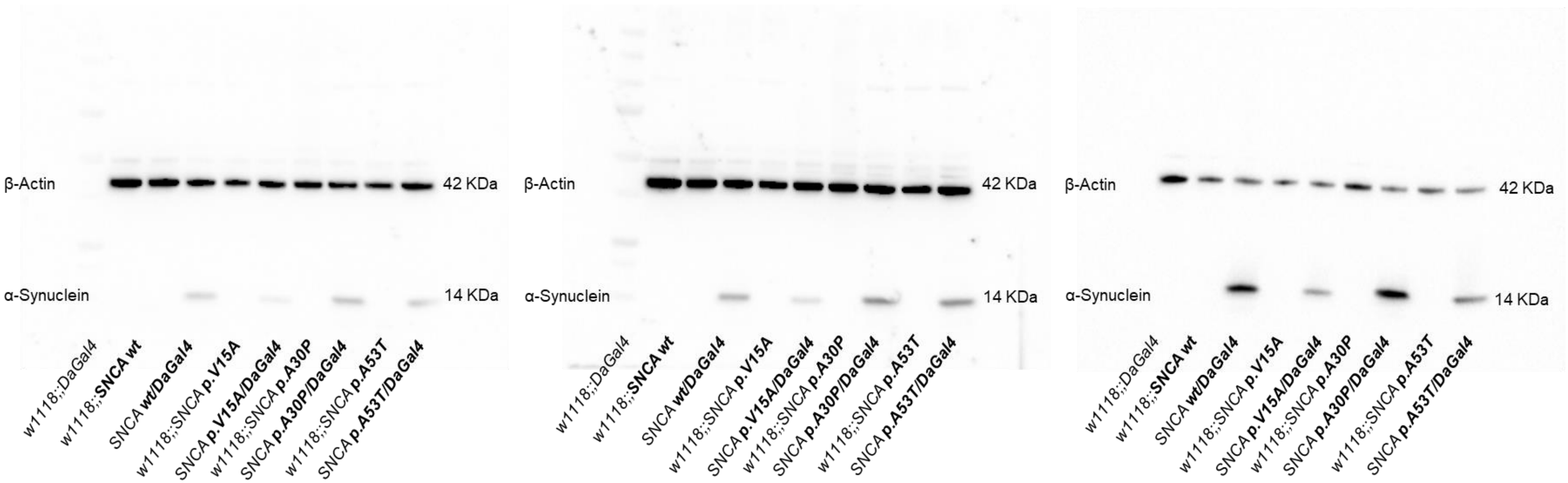


**Uncropped western blots corresponding to Figure 6: Parkinsonian-like phenotypes in flies overexpressing different human *SNCA* variants.**

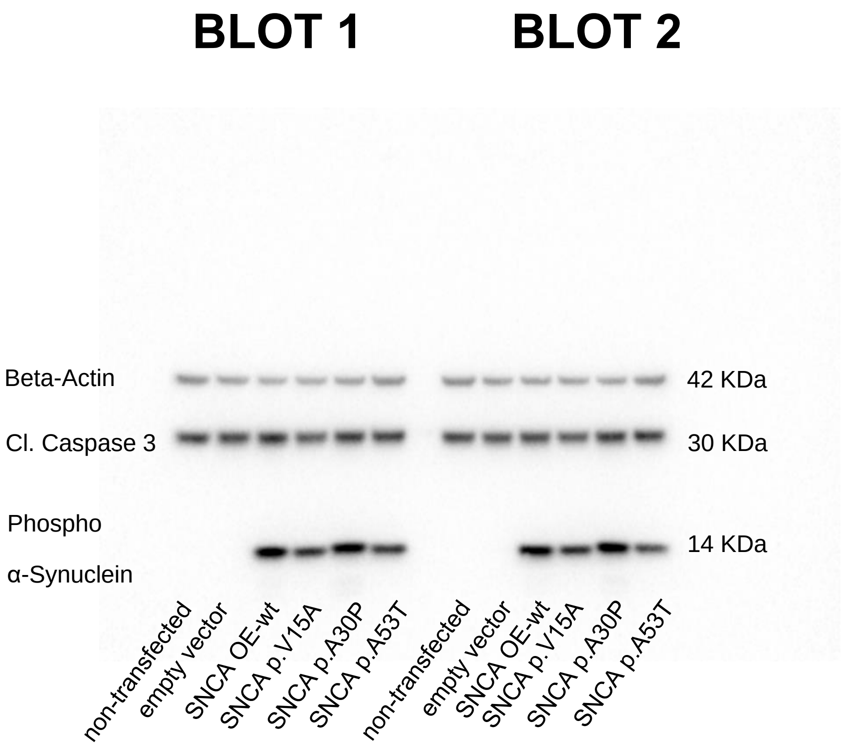


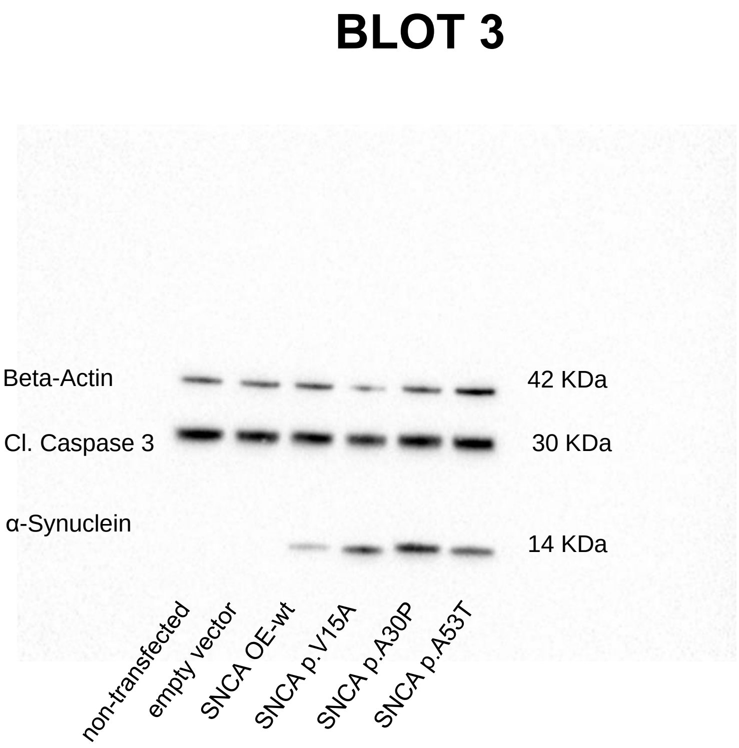


**Uncropped western blots corresponding to Supplemental figure 2.**
