# Supplementary material for: Characterization of the pathogenic α-Synuclein Variant V15A in Parkinson´s disease
Source: NPJ Parkinsons Dis. 2023 Oct 30;9:148. doi: 10.1038/s41531-023-00584-z (PMC10616187; doi:10.1038/s41531-023-00584-z)
Supplement: Supplementary file 2 — Reporting summary [file 41531_2023_584_MOESM2_ESM.pdf]

nature portfolio

Corresponding author(s): Katja Lohmann

Last updated by author(s): Aug 4, 2023

Reporting Summary

Nature Portfolio wishes to improve the reproducibility of the work that we publish. This form provides structure for consistency and transparency in reporting. For further information on Nature Portfolio policies, see our Editorial Policies and the Editorial Policy Checklist.

Statistics

For all statistical analyses, confirm that the following items are present in the figure legend, table legend, main text, or Methods section.

n/a	Confirmed	
☐	☒	The exact sample size (*n*) for each experimental group/condition, given as a discrete number and unit of measurement
☐	☒	A statement on whether measurements were taken from distinct samples or whether the same sample was measured repeatedly
☐	☒	The statistical test(s) used AND whether they are one- or two-sided<br>*Only common tests should be described solely by name; describe more complex techniques in the Methods section.*
☒	☐	A description of all covariates tested
☐	☒	A description of any assumptions or corrections, such as tests of normality and adjustment for multiple comparisons
☐	☒	A full description of the statistical parameters including central tendency (e.g. means) or other basic estimates (e.g. regression coefficient) AND variation (e.g. standard deviation) or associated estimates of uncertainty (e.g. confidence intervals)
☐	☒	For null hypothesis testing, the test statistic (e.g. *F*, *t*, *r*) with confidence intervals, effect sizes, degrees of freedom and *P* value noted<br>*Give P values as exact values whenever suitable.*
☒	☐	For Bayesian analysis, information on the choice of priors and Markov chain Monte Carlo settings
☒	☐	For hierarchical and complex designs, identification of the appropriate level for tests and full reporting of outcomes
☐	☒	Estimates of effect sizes (e.g. Cohen's *d*, Pearson's *r*), indicating how they were calculated

Our web collection on statistics for biologists contains articles on many of the points above.

Software and code

Policy information about availability of computer code

Data collection	The measurement of mRNA levels was obtained using LightCycler 96 System Software (Roche).<br>Western blot figures were collected using Image Lab Software (Bio-Rad).<br>Native Dot blot figures were collected using LI-COR Image Studio Lite Software (Li-cor).<br>Immuno-staining images were obtained using ZEN Microscopy Software (Zeiss).
Data analysis	Western blot analysis was performed using Image Lab Software (Bio-Rad).<br>Native Dot blot figures were analysed using LI-COR Image Studio Lite Software (Li-cor).<br>Form factors were calculated by using ImageJ (NIH software).<br>All statistical analyses were performed using GraphPad Software (Prism).

For manuscripts utilizing custom algorithms or software that are central to the research but not yet described in published literature, software must be made available to editors and reviewers. We strongly encourage code deposition in a community repository (e.g. GitHub). See the Nature Portfolio guidelines for submitting code & software for further information.

Data

Policy information about availability of data

All manuscripts must include a data availability statement. This statement should provide the following information, where applicable:

- Accession codes, unique identifiers, or web links for publicly available datasets
- A description of any restrictions on data availability
- For clinical datasets or third party data, please ensure that the statement adheres to our policy

The datasets used and analysed during the current study are available from the corresponding author upon reasonable request, i.e., for research purposes by respectively qualified individuals.

Research involving human participants, their data, or biological material

Policy information about studies with human participants or human data. See also policy information about sex, gender (identity/presentation), and sexual orientation and race, ethnicity and racism.

Reporting on sex and gender	The studied patient was a male who had an affected but deceased mother. For functional characterization, four male and four female individuals were used. The findings are applicable to both sexes, as sex was deliberately considered during the selection process to create a diverse and representative population. Participants' sex was determined through self-reporting.
Reporting on race, ethnicity, or other socially relevant groupings	The patient was of Turkish origin. This was given and not relevant for the study design.
Population characteristics	Four healthy control participants, aged 57, 63, 63, and 74, provided biological samples during the study. Additionally, four participants with an SNCA genotype carrying a pathogenic variant and diagnosed with Parkinson's Disease donated biological material. The ages of the affected participants were 46, 48, and 51 (two participants).<br><br>Patient 1, a 48-year-old male of Turkish origins living in Germany, experienced disease onset at the age of 40. The treatment regimen for Patient 1 included levodopa/carbidopa/entacapone (150/37.5/200 mg) five times a day, safinamide 50 mg daily, and clozapine 12.5 mg per day.
Recruitment	The patient was collected in the outpatient clinic. The controls and other patients were collected at the same institution (in the framework of research projects).
Ethics oversight	The ethics committee of the University of Lübeck approved the study.

Note that full information on the approval of the study protocol must also be provided in the manuscript.

Field-specific reporting

Please select the one below that is the best fit for your research. If you are not sure, read the appropriate sections before making your selection.

☒ Life sciences ☐ Behavioural & social sciences ☐ Ecological, evolutionary & environmental sciences

For a reference copy of the document with all sections, see nature.com/documents/nr-reporting-summary-flat.pdf

Life sciences study design

All studies must disclose on these points even when the disclosure is negative.

Sample size	We established six distinct SH-SY5Y neuroblastoma cell models (n=6): a non-transfected line, a line with an empty vector, and lines overexpressing the variants p.V15A, p.A30P, p.A53T, and the wildtype.<br><br>In addition, we generated and characterized iPSC lines from four control individuals, three patients with the p.A53T mutation, and one patient with the p.V15A mutation. The iPSCs were directly differentiated into dopaminergic neurons. The control neurons were collected at day 86 (n=4) and day 100 (n=4), while patient-derived neurons were harvested at day 90 of differentiation (n=4).<br><br>For the Drosophila model study (n=9), we included samples with ubiquitous expression using the DaGal4 system and compared them to no expression when the driver was absent. We acquired SNCA wildtype, SNCA p.A30P, SNCA p.A53T, w1118, and daughterlessGal4 (DaGal4). Additionally, SNCA p.V15A was generated by cDNA insertion.<br><br>The sample size was determined considering various factors, including availability, practical constraints, and complexities involved in generating the different lines.
Data exclusions	No data were excluded.
Replication	The study protocol, including the experimental design, methods, and procedures, was thoroughly documented to ensure transparency and

Replication	facilitate replication. Data were collected, processed, and analyzed using the same techniques multiple times to determine if the results aligned with the original findings.All attempts at replication were successful.
Randomization	To minimize the impact of confounding factors, participants with similar characteristics were paired or matched (I.e. same variant/genotype/disease condition).Subgroup analyses were performed to explore potential effect modifications by certain covariates. This allowed to examine whether the treatment effect varies among different subgroups based on specific characteristics.
Blinding	Blinding was not possible and relevant to this study since we performed a non-randomized allocation method, and the allocation was based on specific characteristics or criteria (genotype). Other measures have been implemented to minimize bias and ensure the study's validity, such as employing objective outcome measures and conducting rigorous data analysis and interpretation.

Reporting for specific materials, systems and methods

We require information from authors about some types of materials, experimental systems and methods used in many studies. Here, indicate whether each material, system or method listed is relevant to your study. If you are not sure if a list item applies to your research, read the appropriate section before selecting a response.

Materials & experimental systems

n/a	Involved in the study	
☐	☒	Antibodies
☐	☒	Eukaryotic cell lines
☒	☐	Palaeontology and archaeology
☐	☒	Animals and other organisms
☐	☒	Clinical data
☒	☐	Dual use research of concern
☒	☐	Plants

Methods

n/a	Involved in the study	
☒	☐	ChIP-seq
☒	☐	Flow cytometry
☒	☐	MRI-based neuroimaging

Antibodies

Antibodies used	anti-α-Syn antibody (1:1000 #2647; Cell Signaling)<br>anti-Cl. Caspase 3 (1:1000, #9662; Cell Signaling)<br>anti-β-Actin (1:1000000 #A5316; Sigma)<br>anti-Rabbit IgG, HRP-linked antibody (1:2500; Cell Signaling)<br>anti-Mouse IgG, HRP-linked antibody (1:2000; Cell Signaling)<br>MJFR-14-6-4-2 (1:2000 #ab209538; Abcam)<br>IRDye 800CW Goat anti-Rabbit (1:20000; Li-COR)<br>anti-GRP75 (1:1000 # ab53098; Abcam)<br>Goat anti-Rabbit IgG Alexa Fluor 488 (1:400; Invitrogen)
Validation	When possible the antibodies were validated using a negative control.

Eukaryotic cell lines

Policy information about cell lines and Sex and Gender in Research

Cell line source(s)	We established six distinct SH-SY5Y neuroblastoma cell models (n=6): a non-transfected line, a line with an empty vector, and lines overexpressing the variants p.V15A, p.A30P, p.A53T, and the wildtype.<br><br>In addition, we generated and characterized iPSC lines from four control individuals, three patients with the p.A53T mutation, and one patient with the p.V15A mutation. Skin fibroblasts were reprogrammed into iPSCs and directly differentiated into dopaminergic neurons. Four healthy control participants, aged 57, 63, 63, and 74, provided biological samples for neuronal differentiation. Additionally, four participants with an SNCA genotype carrying a pathogenic variant and diagnosed with Parkinson's Disease donated biological material. The ages of the affected participants were 46, 48, and 51.
Authentication	All line were established in-house. relevant pathogenic variants were confirmed by Sanger sequencing
Mycoplasma contamination	All cell line tested negative for mycoplasma contamination.
Commonly misidentified lines<br>(See ICLAC register)	not applicable

Animals and other research organisms

Policy information about studies involving animals; ARRIVE guidelines recommended for reporting animal research, and Sex and Gender in Research

Laboratory animals	Drosophily melanogaster
Wild animals	None
Reporting on sex	Sex had no impact on our studies
Field-collected samples	Not applicable
Ethics oversight	Not needed

Note that full information on the approval of the study protocol must also be provided in the manuscript.

Clinical data

Policy information about clinical studies

All manuscripts should comply with the ICMJE guidelines for publication of clinical research and a completed CONSORT checklist must be included with all submissions.

Clinical trial registration	this is not a clinical trial
Study protocol	this is not a clinical trial
Data collection	Neurological examination of the patients in the diagnostic context
Outcomes	Not applicable
